# Seeing the unseen: problematic narratives and the microbial worlds of the deep-sea

**DOI:** 10.1007/s40656-025-00682-6

**Published:** 2025-07-01

**Authors:** Teun Joshua Brandt

**Affiliations:** Department of European Literature and Culture and Department of Theoretical Philosophy (Interfaculty Position), University of Groningen, Groningen, The Netherlands

**Keywords:** Deep-sea science, Cultural narratives, Deep-sea microbes, Cultural imagination

## Abstract

Microbes emerge as the protagonists of new scientific narratives of the deep-sea, functioning as life-givers in biological communities or central actors in geochemical cycles essential to the functioning of the Earth system. This paper aims to address the significant discrepancy between present-day scientific knowledge and popular narratives of the deep, with a particular focus on the narrative representation of deep-sea microbes. In the face of looming deep-sea ecocide, it is crucial to incorporate knowledge of the microbial ‘unseen majority’ into our ecological imagination and challenge the ‘size bias’ towards larger organisms. However, problematic narratives—such as portraying the deep-sea as a barren place, as a place full of alien life, or as the last frontier on earth—persist in the cultural imagination and deflect profound engagement with deep-sea microbial worlds. I discuss Frank Schätzing’s novel *Der Schwarm* (2004) as a hopeful exception, proving that a profound engagement with deep-sea worlds does not defy the dynamics of popular fiction. Instead of recycling old storylines, the novel articulates the relevance of deep-sea ecosystems to the Earth system and positions the deep-sea microbe as an important actor in human lifeworlds instead of letting it reside at the fringes of the cultural imagination.

## Introduction

We have explored only a small fraction of Earth’s oceans, and most of that knowledge lies in shallow waters, while the depths beneath remain a mystery.^1^ Exploring the deep-sea^2^ requires highly advanced technology that was not available until recently. Due to this inaccessibility, the deep-sea has remained a long-standing mystery, sparking the imaginations of scientists and novelists alike. Whether viewed as a barren desert devoid of life, as consisting of a ‘primordial soup,^3^’ or as a realm inhabited by fantastical creatures, the deep-sea stands as a paradigmatic example of a *storied place*—a place historically conjured by the imagination.^4^ Starting in the late twentieth century, however, an accumulation of discoveries began to shake the foundations of enduring scientific and cultural narratives. In 1977, scientists aboard the American submersible *Alvin* made a groundbreaking discovery when they encountered what they termed ‘black smokers,’ now recognized as hydrothermal vents. Contrary to earlier expectations, these vents revealed a surprising abundance of life, defying the odds posed by the absence of sunlight, immense pressure and temperatures soaring up to four hundred degrees Celsius. Over the following decade, further exploration uncovered other deep-sea habitats, such as whale falls and cold seeps, fundamentally transforming our understanding of biodiversity in the deep ocean. While it was once believed that organic detritus from the surface ocean, also known as ‘marine snow,’ was the sole energy source sustaining deep-sea life, these discoveries revealed that many organisms can thrive in these extreme environments thanks to their symbiotic relationships with chemosynthesising microorganisms. These microbes convert carbon-containing molecules into energy, supporting large biomass and unique, highly diverse ecosystems in otherwise food-limited environments. These deep-sea microbes not only constitute the bedrock of unique ecosystems but also serve as harbingers of groundbreaking insights into the origins of life on our planet^5^ while simultaneously expanding our understanding of the possibilities for life on other planets.^6^ Abyssal plains, in turn, the vast slopes between oceanic ridges and continental margins once thought to be lifeless deserts, revealed an unexpected species diversity that may rival that of rainforests, with most of these species being microscopic (Ebbe et al., 2010; Grassle & Maciolek, 1992). As a result of these ground-breaking discoveries, deep-sea microbes emerge as central figures in the evolving scientific narratives of the deep, serving as life-givers in biological communities, key actors in geochemical cycles vital to the Earth system, and precursors holding answers to some of the most focal questions about life.^7^

Despite significant advancements in deep-sea science, outdated and problematic narratives continue to persist in the popular scientific imagination. This article aims to highlight this gap between scientific discoveries and popular narrative representations of deep-sea worlds, with the deep-sea microbe serving as a case study emblematic of broader issues in the narrative representation of life beyond our immediate sight. The first section of this article explores three prevailing narratives about the deep-sea: the deep as *a barren place*, *a realm full of alien life*, and *the last wilderness/frontier on Earth*, with particular emphasis on how these narratives intersect with, draw upon, or are reinforced by the microbial worlds of the deep. Throughout the analysis, I will draw on examples from both popular culture and scientific discourse, emphasizing that these narratives, far from being confined to the arts or lay discourse, function as some sort of ‘memes’^8^—small units of semiotic articulation (i.e., a way of meaning-making)—that easily circulate across various materials and discourses, carrying their inherent affordances with them.^9^ In light of expanding scholarship on narrative in science (Beer, 2009; Morgan et al., 2022; Newman, 2022), I recognize the crucial role of such narratives in how scientists, as Stefan Helmreich puts it, seek to find meaning in the life they study, looking for a “message from the mud” (2009, p. 31). The term ‘popular scientific imagination,’ then, refers to a shared space of meaning-making that not only highlights the narrative dimension of scientific knowledge making and communication but also extends beyond the boundaries of scientific knowledge production, which is itself shaped by the cultural and societal contexts and modes of meaning-making in which it operates, including those of popular science and science fiction writing (with the primary difference arguably being the treatment of factuality). The ocean is “a natural as well as cultural object,” Stefan Helmreich writes, “a material thing that becomes meaningful only through perception, belief, and action” (2009, p. X). Likewise, science communication, popular science, and science fiction cannot escape the pull of narrativity which we are so familiar with, storying the ‘mud’—to borrow Helmreich’s reference to the troubled waters of deep ocean science—into comprehensive, compelling but often also simplifying and problematic narratives. In this regard, the three narratives of the deep outlined in this article serve as an initial taxonomy for further investigation. To fully understand their (historical) prevalence across media, further quantitative analysis will be necessary—a task that lies beyond the scope of this article.

I place ‘historical’ in parentheses because, while these narratives may be observed independently and have gained or lost traction over time, they continue to widely capture the popular scientific imagination of the deep and are often found together, as illustrated by the opening line of an informational online video on the deep-sea: “There is still one place to explore. It’s a wet and deadly desert inhabited by mysterious creatures living in total darkness” (Kurzgesagt, 2019, p. 12). The paradox in this—that we are only beginning to ‘explore’ the deep-sea yet already story what lies beneath—underscores the deep ocean in particular as a site of cultural construction, reflecting a rough continuum where the upper, light-filled zones of the ocean are seen as areas of greater scientific ‘illumination,’ while the deeper one goes, the less ‘scientific light’ penetrates and the more ‘storied’ it becomes. Yet, as will be discussed, this metaphor—and, more broadly, the framing of the deep as the ‘last scientific frontier’—carries the cultural baggage of, among other things, portraying the deep as a ‘wild’ place outside of human history. Especially in our current era, characterized by the rapid acceleration of global industrialization and the disruption of biogeochemical cycles—pushing Earth’s systems toward increasingly unpredictable and precarious tipping points—such a narrative becomes untenable as we find ourselves ever more entangled in the complex relationships of the Earth system (Bonneuil & Fressoz, 2016, p. 20). As the deep-sea plays an important role in these complex relationships, with what kind of stories we construct this ‘storied’ place matters. Given recent developments in deep-sea mining, which push the commodity frontier into uncharted depths, the stories we tell about the deep-sea are no longer (if they ever truly were) merely descriptive or representational; they have become profoundly consequential. As major environmental disruptions such as ecocide and species extinction loom, the long-standing narrative of the deep-sea as a barren place may transform into reality before it fully takes hold as part of the historical vernacular. Despite this, deep-sea life is rarely regarded as pivotal in environmental narratives in comparison to shallow waters (Jamieson et al., 2021; Vincent, 2011), even though research demonstrates its direct relevance to human life-worlds (La Bianca et al., 2023; Souster et al., 2024; Thurber et al., 2014). Similarly, despite their crucial role in the Earth system, the ‘unseen majority’ of microorganisms remains largely neglected within the environmental movement (Berti et al., 2020; Cockell, 2008). This dual neglect relegates deep-sea microbes to the peripheries of our environmental imagination, casting them as paradigmatic figures of difference and, consequently, fostering indifference. Popular culture, meanwhile, fixates on giant sharks and kraken—ultimate monstrosities haunting us from the depths—reinforcing the perception of the deep as a realm teeming with alien life, distant from us both in space, taxonomy, and perceived relevance to terrestrial ways of life. Therefore, comprehending the vital roles played by this ‘unseen majority’ of deep-sea microbes in the broader ecological framework is not only imperative for marine science but also increasingly crucial in the stories we tell.

In the second part, I will discuss Frank Schätzing’s novel *Der Schwarm* (2004) as a hopeful example that incorporates current knowledge of the deep-sea, critically engages with problematic narratives and faces the anthropogenic ecocide at sea. In the novel, a hyperintelligent, multicorporeal, and microbial threat from the deep-sea arises to defend itself against the anthropogenic destruction of the oceanic habitat. Instead of recycling old storylines, the novel articulates the relevance of deep-sea ecosystems to the Earth system and positions the deep-sea microbe as an important actor in human lifeworlds rather than relegating them to the fringes of the cultural imagination—demonstrating that a profound engagement with microbial deep-sea worlds does not defy the dynamics of popular fiction.

## Deep-sea narratives

### A barren place

The deep-sea was, and still is, perceived as a barren place—*terra nullius*, indicative of a lack of life—clearing the way for its exploration, invasion, dispossession, or even destruction. This narrative is primarily based on the azoic zone theory proposed by Edward Forbes in the mid-nineteenth century. According to this theory, biodiversity decreases with depth, suggesting that life cannot thrive beyond approximately 600 m (Anderson & Rice, 2006; Jannasch & Taylor, 1984, p. 487), and continued to be envisioned by many biologists (e.g., Ekman, 1953; Williams, 1964). During the famous 1977 Alvin dive that discovered hydrothermal vents, geologist Jack Corliss asked, “Isn't the deep ocean supposed to be like a desert?” (WHOI, 2025). Although the theory of the deep as a lifeless desert has been conclusively debunked by the discoveries of cold seeps, whale falls, and hydrothermal vents, it persists as a narrative in relation to abyssal plains. “At one time,” Scheckenbach et al. write, “these plains were assumed to be vast, desert-like, contiguous habitats” (2010, p. 115). Recent discoveries are gradually reshaping our perspective on abyssal plains, revealing ‘higher-than-expected’ species richness and heterogeneity (Ramirez-Llodra et al., 2010; Sigwart et al., 2023, p. 2), serving as significant contributors to essential ecosystem services (Simon-Lledó et al., 2023). Additionally, new studies emphasize the high susceptibility of abyssal plain life to anthropogenic influences, including climate change (Levin et al., 2020; Sweetman et al., 2017) and deep-sea mining (Jones et al., 2017; Simon-Lledó et al., 2019), which reminds us that portraying the deep-sea biome as desert-like and thereby implying that it is life-poor can be dangerous—especially when it legitimizes extraction practices with consequences that are not yet fully understood or known but potentially devastating.

Following recent discoveries demonstrating an abundance of life in various deep-sea locations, another narrative has emerged within the scientific community: that of vast barren plains interspersed with islands thriving with life. Marine geologist Furu Mienis states, for example, that “the deep-sea, in most places, is *barren* and flat. And then, suddenly, we have these sponge grounds that form colourful, thriving communities. It is intriguing how this system sustains itself in such a place” (NIOZ, 2021). This narrative manifests in different iterations elsewhere (e.g., R. Fisher, 2020; Vrijenhoek, 2002) and has the potential to perpetuate the myth of a barren ocean desert, making it susceptible to exploitation as a greenwashing argument. Such exploitation could involve advocating for the preservation of specific areas while simultaneously justifying the mining of other regions to support the energy transition. Reserving abundant sites such as hydrothermal vents or cold seeps for protection alone, as has been suggested (Van Dover, 2014), not only conflicts with the known (and potential) ecosystem services of abyssal plain life (Orcutt et al., 2020) but also may overlook the interrelation between different biomes, especially in regard to the consequences of deep-sea mining.

A key factor sustaining the narrative of deep-sea deserts is that the majority of life at ocean depths is microbial, remaining hidden in plain sight. As they lack ‘phenomenal resolution’,^10^ they easily elude the human imagination. The ‘unseen majority’ of microbial life remains largely unstudied and unregarded within the environmental movement compared to ‘charismatic megafauna’ such as whales or dolphins (Berti et al., 2020). In general, the protection of endangered species typically focuses on large mammals or birds, as noted by Charles S. Cockell: “Environmental policy has a size bias” (Cockell, 2008, p. 23). It might, therefore, not be a mere coincidence that abyssal plains persist as one of the least explored environments on the planet, lacking any vertebrate megafauna.

### A place full of alien life

Since early human civilizations, the depths of the open ocean have evoked images of ancient monstrous mythological creatures such as sea serpents, the Leviathan, and the Kraken. “The open sea has been subject to our fascination since the earliest attempts at seafaring,” Harrington observes, as its enigmatic nature prompts unsettling reflections “on the precarious nature of the boundaries of knowledge” (2018, p. 27). These boundaries allow for cultural fantasies featuring figures of alterity and the unknown—and alterity is crucial, as most mythical sea creatures appear alien, monstrous, or otherworldly. Consider the giant ‘poulps’ of Jules Verne's *Twenty Thousand Leagues Under the Seas*, a quintessential work of deep-sea literature, or H.P. Lovecraft’s monstrous figure of Cthulhu. In more recent popular culture, we find a giant eurypterid in *DeepStar Six* (1989), mutant creatures in *The Rift* (1990), giant electric eels in *Deep Schock* (2003), a supernatural sea monster in *The Evil Below* (1989), a man-sized stingray-like mutant in *Lords of the Deep* (1989), a lamprey-like mutant in *Leviathan* (1989). In the twenty-first century, the trend continues, presenting us *The Shape of Water* (2017) by Guillermo del Toro, in which the protagonist falls in love with a humanoid amphibian creature; *Underwater* (2020) by William Eubank, which is an adaptation of Lovecraft’s Cthulhu story; or *The Meg* (2018), in which a prehistoric and monstrous shark emerges from the depths. The fascination for the deep-sea alien has entered the age of online video platforms such as *YouTube* and generated many clickbait-titles such as “15 Mariana Trench Creatures That Are Scarier Than Megalodon” (Amerikano) or “The Most Terrible Deep-sea Creatures You've Never Seen Before” (BRAIN TIME). All these stories converge on a common plotline, succinctly summarized by Eugene Thacker: “The abyss of the unknown sea reaching up to the surface with a certain inevitability” (2015, p. 370). All the fictional creatures in these stories are, in some sense, reconfigurations of the same monstrous figure haunting humanity from the (metaphorical *and* literal) abyss—serving as embodiments of humanity’s fears of the unknown.

Contrary to the gargantuan monsters of fictional imagination, figurations of alterity are not always monstrous horrors; they may be microscopic and arise from a sense of (scientific) wonder rather than fear. In *Alien Ocean*, Stefan Helmreich discusses how marine biologists draw on the figure of the ‘alien’ to characterise the microbial peculiarities of marine life, signifying the strangeness of hydrothermal vent microbes or the potential of marine microbes as models for extraterrestrial life (2009, p. XI). Helmreich underscores the close connection between scientific narratives and those in popular culture, emphasizing the exchange of tropes, plotlines, and motifs, in imagining life in the deep (2009, p. 16, p. 66, p. 75). In this context, taking James Cameron’s 1989 science fiction movie *The Abyss* as a typical example, Helmreich illustrates how the figure of the alien functions in both scientific and popular culture as a mirror for human self-conception and societal anxieties (p. 16). However, the alien-as-mirror motif also highlights the need for caution in narratives portraying the deep as a realm of alien or monstrous life, as the mirror (i.e., the alien) fosters an illusion of absolute opposition, reinforcing folk-biological taxonomies that shape ethical judgments. In other words, the problem of depicting deep-sea lifeforms as ‘alien’ and monstrous creatures lies in its supposed difference, and because “that what is beyond the self-defined differentiating border of comfort (*difference*) is socially made legitimate to be neglected (*indifference*)” (Van Houtum & Van Naerssen, 2002, p. 129). In particular, this issue becomes a problem of ethical taxonomy in the context of microbial worlds, as discussed by Barbara Herrnstein Smith:

When the issue is our responsibility to others, questions about limits are inevitably complicated by questions about sorts, and the relation between them broaches a domain we might call ethical taxonomy. Should we, for example, have care for dogs, cats, cows and horses but not birds, snakes or butterflies? For leopards and walruses but not lobsters or oysters? For all these, but not wasps, tick or lice? Or for these, too, but not microbes or viruses? (2004, p. 2)

According to Smith, classifications are not “abstract, neutral, inert containers” (2004, p. 3). Instead, the way we relate to animals is heavily influenced by our bodily inclinations. Practically, this means that we see certain animal species, foremost mammals, as kin or kind and other species such as insects, invertebrates or microorganisms as “alien or remote” (2004, p. 5). The representation of deep-sea lifeforms as ‘alien’ others, figures of alterity, not only articulates the idea that they appear to us as different beings but also trivializes their importance to human lifeworlds. This is certainly the case in regard to the understanding of deep-sea microbial worlds: As they are tiny, not perceivable for the human eye, residing in a different biological kingdom than we do, inhabiting a remote region inaccessible to us terrestrial beings, they are regarded as fundamentally different and therefore neglectable.

According to Jennifer A. Mather, the environmental imaginary is heavily biased by a focus on mammals. The ‘value’ we place on life forms is not based on “species number or ecological importance but on our social cognition and the way in which we understand and position them” (2019, p. 2). As Maureen O’Malley points out, “we usually feel it is tragic when extinction happens to orchids, birds or frogs, but such sentiments are rarely in evidence for microbes” (2014, p. 203). This is problematic, Mather writes, as this value influences ‘political power’ by pressure of interest groups (NGOs), who are mostly concerned with large megafauna. “As one goes ‘down the phylogenetic tree’,” Mather writes, “vertebrates appear less and less like humans and thus their appeal wanes” (2019, p. 3). The negative viewpoint of the so-called ‘lower’ life-forms starts in childhood, and “experts fuel the public’s biases, which are then fed back to us” (ibid.). Unfortunately, Mather only propagates care and concern for other animals that also feel pain, such as fish or cephalopods. This is problematic in two ways: it ignores the value of nonanimals that do not feel pain, as far as we know or can tell, and it neglects the ecological interdependencies between different life forms: ‘lower’ creatures such as microbes or insects, as well as fungi, plants and protists are essential for ecosystems on which ‘higher’ pain-feeling animals depend upon. This is especially true for deep-sea habitats: deep-sea microbes are not only quintessential for the proliferation of deep-sea organisms (McFall-Ngai et al., 2013) but also crucial for sustaining life in other ecosystems (Thurber et al., 2014), highlighting the complexity of interdependence and the entanglement of species across different biomes *and* taxa.

Displaying deep-sea lifeforms as ‘aliens’ aligns with the more general sea/land division. The term terrestrial life has an ambiguous meaning, as it is used in opposition to marine life on the one hand and to extraterrestrial life/alien life on the other. This ambiguity reinforces the supposed equation of aquatic life to extraterrestrial/alien life. Although the sea is unquestionably part of nature, Håkan Sandgren writes in *Life Under Water*, it is also “an environment foreign or even hostile to humans, … a counternature when seen from an anthropocentric perspective” (2017, p. 260). And perceiving the sea as a counternature, he writes, “has made it easier for us to use it in an ill-considered and reckless way, as a dumping ground for waste, as an infinite tank for pollutants, and as a seemingly inexhaustible supply of food resources” (2017, p. 261). While the upper zones of the sea can still be considered transitional in the land/sea division and play more active roles in human lifeworlds, the deep-sea stands out as a nonterrestrial ‘counternatural’ place and, as a result, as a paradigmatic place of indifference. Consider this bizarre 1991 article in the New York Times:

Black as a cave, *devoid of plants* and *home to worms and other low forms of life* [emphasis added], the thick sediments at the bottom of the deepest oceans are described by marine scientists as immense and *empty plains* [emphasis added] undisturbed by earthquakes or volcanoes. Now scientists at the Woods Hole Oceanographic Institution, one of the nation's most respected marine research centers, believe they have found a use for what they call the planet’s most useless real estate … dumping sewage sludge. (Schneider, 1991)

This article synthesises previously discussed narratives into a proposal advocating for the use of the deep-sea as a global wastebin, which, from contemporary scientific perspectives highlighting the proliferation and significance of deep-sea ecosystems, appears absurd. The article, however, functions as a poignant reminder of the dangers of simplistic narratives—especially those that create the illusion of knowing and understanding—ultimately obscuring the crucial acknowledgement that the deep-sea still largely remains a ‘dark space of knowledge.’ This reminder is vitally relevant at this time, as industries are increasingly turning their attention to the seafloor for mining opportunities, aiming to stretch this last ‘commodity frontier,’ while the impact is largely unknown but potentially devastating. In the face of this, environmentalists are increasingly concerned with preserving this ‘last unspoiled wilderness.’ This brings me to the final narrative.

### The last wilderness/frontier on earth

In the Age of the Anthropocene, the idea of wilderness, a place contrary to human civilization, seems out of date: it is estimated that 84 per cent of the ice-free land surface of the planet is under direct human influence (Ellis, 2011). However, as William Cronon argues, precisely because the border between human influence and the natural has become messy, a deep fascination for remote ecosystems that are ‘left alone’ fills our imagination (1996, p. 82). Wilderness serves, he writes, as the foundation concept upon which many environmentalist narratives are being built (1996, p. 80). The deep-sea, a place imagined to be remote enough to stay out of human interference, has become one of them.

The idea of wilderness, as we know it today, is of very recent fashion. “Go back 250 years in American and European history,” Cronon writes, “and you do not find nearly so many people wandering around remote corners of the planet looking for what today we would call ‘the wilderness experience’” (1996, p. 70). In contrast, what was thought of to be wilderness was by no means ‘pristine’ but rather meant ‘deserted,’ ‘desolate,’ ‘barren.’ Within literary studies, this is usually referred to as *locus terribilis*, ‘terrible place.’ By the end of the nineteenth century, however, the wild became more and more a *locus amoenus*, ‘pleasant place.’ Whereas wilderness “had once been the antithesis of all that was orderly and good—it had been the darkness, one might say, on the far side of the garden wall—and yet now it was frequently likened to Eden itself,” Cronon writes (1996, p. 72). The deep-sea is a paradigmatic example of this: initially, it was perceived as dangerous, dark, and devoid of life. However, as scientific discoveries ‘enlightened’ the darkness of the deep, it began to emerge as a pristine wilderness full of life—a ‘Garden of Eden,’ with the first vent discovered aptly named accordingly (WHOI, 2025).

The concept of wilderness is intricately linked to that of the frontier, where the wilderness signifies the uncharted space beyond this boundary (Cronon, 1996, p. 77). The notion of ‘the last frontier,’ however, has two possible readings, both concerned with its expansion. On the one hand, it can be understood as a ‘commodity frontier’—a concept coined by Jason W. Moore to describe the incorporation of areas and places previously external to the capitalist world economy (2000). On the other hand, it can be perceived as a ‘scientific frontier,’ denoting the demarcation between the known and the unknown.

These readings converge in the deep-sea, where scientific revelations have attracted mining interests, especially with the discovery of valuable deposits such as manganese and cobalt, which are crucial for meeting the increasing demand for batteries. Mining companies assert the need for these minerals to sustain the energy transition and emphasize the feasibility of ‘sustainable’ deep-sea mining, highlighting contained impacts within the boundaries of the deep-sea. However, scientists caution against this, arguing that deep-sea ecosystems remain inadequately understood, with emerging studies indicating potential widespread disruption from mining activities (Halfar & Fujita, 2007; Simon-Lledó et al., 2019; Souster et al., 2024; Stenvers et al., 2023). Additionally, scientists repeatedly criticize the technoscientific narrative of sustainable deep-sea mining, as it is misleading and obscures possible environmental impacts (cf. EASAC, 2023; Hallgren & Hansson, 2021). If future studies confirm that deep-sea mining exacerbates climate change—supported by current research indicating that disturbing the seabed drastically diminishes the carbon capture services of deep-sea life (Sala et al., 2021; Souster et al., 2024)—the concept of sustainable deep-sea mining becomes a paradox demanding critical attention.

This tension has fuelled an environmental movement advocating for the protection of the deep from extractivist industries. Greenpeace and the United Nations actively champion the preservation of what they refer to as “the last remaining ocean wilderness” (Brooks, 2023; Davis, 2018; McCabe, 2023). That the deep-sea is a last remaining wilderness is, however, more of a desire than a reality, as several studies show (Ramirez-Llodra et al., 2010; Thiel, 2003). Its remoteness, Thiel writes, “has not protected the deep-sea from anthropogenic impacts. Wastes released anywhere on the high seas or into the atmosphere may rapidly sink into the abyss, to be out of sight and out of mind” (Thiel, 2003, p. 427). Islands of plastic are appearing throughout the deep (Mohrig, 2020), and the tracks of mining vessels have already left barren marks, even though deep-sea mining is still in its infancy (Fisher, 2020).

## The Swarm

Through its critical engagement with the problematic narratives discussed so far, its incorporation of up-to-date scientific knowledge, and its framing of climate change and ecological destruction, Frank Schätzing’s novel *Der Schwarm* (2004)^11^ serves as a compelling example of how narrative *can* play a valuable role in shaping the environmental imagination of the deep. From the outset, the novel highlights the anthropogenic impact on the seas. In the prologue, we are introduced to the story of a Peruvian fisherman, one of the last of his kind, who tells us that the sea, once teeming with fish and capable of sustaining an entire population of fishermen, has now become a lifeless void: “‘There was a desert here once,’ his father had told him, ‘the desert plains. Now we’ve got two deserts—the plains and the ocean beside them. We’re desert-dwellers threatened by rain’” (Schätzing, 2006, p. 8). This sets the tone for the following section, where a series of characters and conversations introduce the current stakes and narratives surrounding the deep. Among them is Johanson, a marine biologist and professor at the University of Trondheim, who occasionally collaborates with Lund, a representative of Statoil, Norway’s state-owned petroleum company. Lund is a personification of overlapping interests between mining companies and ‘independent’ science within technoscientific imaginings of the deep-sea as a new commodity frontier, as she not only works for Statoil and Sintef, but also for Marintek, a marine technology institute. The Norwegian offshore industry in particular, we learn in the novel, owes its ‘pioneering’ developments to this collaboration—underscoring the delicate balance-act between corporate interests on one hand and concerns about environmental disasters on the other (p. 28). The dangers of ‘stretching this last frontier,’ in turn, are introduced by Dr. Bohrmann, a leading scientist at the research centre Geomar in Kiel. We do not know exactly what is down there, he tells Johanson, “so we can’t recommend drilling” (p. 109). The extractivist industry, however, “will start from exactly the same premise but reach the opposite conclusion” (p. 109). The consequences of stretching this commodity frontier are potentially devastating, Bohrmann tells us: “Every time we tamper with our environment without knowing what we’re doing, we’re dicing with death” (p. 116).

In light of this warning, finding out what *is* down there and what is at stake is a central concern for institutes such as Geomar. However, Bohrmann explains to Johanson that the public is difficult to persuade regarding the institution’s work, particularly due to the prevailing yet disproved narrative that the deep sea is devoid of life and therefore unworthy of closer investigation or conservation:

On the face of it, poking about in sediment or measuring seawater salinity was unlikely to solve the world's problems. Besides, few had any understanding of what the seabed was like. After all, it had taken scientists until the early 1990s to discover the truth. Although it was cut off from the warmth and light of the sun, the bottom of the ocean was not a barren wasteland. Rather, it teemed with life. (p. 100)

This vibrant life is illustrated from the start of the novel, as the motivation for the discussions between Johanson, Lund, and Bohrmann stems from the discovery of a previously unknown species of polychaete worm, which complicates Statoil’s current mining plans. Polychaete worms, we are told, are among the oldest known living creatures (p. 30), living in symbiosis with methane-eating bacteria (p. 33), and while the discovery of a new species is not unusual in deep-sea science, Johanson is tasked by Lund to investigate this case because the unknown species exhibits anomalous numbers on the vast plains of methane hydrate which stabilize many of the continental slopes. As Dr. Bohrmann explains: “They act like cement. If you took away the hydrates, the slopes would look like Swiss cheese. Without the hydrates, there’d be landslides” (p. 104). Given that the newly discovered polychaete worms appear to ‘consume’ the methane hydrates, Bohrmann recalls a climate catastrophe during the Eocene, when the melting of methane hydrates caused the collapse of continental slopes, releasing methane gas into the atmosphere and triggering global warming and climate chaos—highlighting that the deep sea is not a place outside human history but directly connected to terrestrial ways of life.

The relevance of deep-sea interactions with global ecosystems is emphasized in the following sections, where people around the world are attacked by marine animals such as sharks, killer whales, and swarms of jellyfish exhibiting anomalous behaviour that scientists struggle to interpret. Swarms of white deep-sea crabs crawl onto land, spreading a previously unknown virus. Along the coast of Norway, the polychaete worm colonies, introduced earlier, destabilize the continental slope, triggering a catastrophic tsunami. As the novel progresses, a crisis team of scientists and military officers, led by the CIA, urgently works to understand these events and identify the cause of the destruction. Slowly, they make progress in shedding light on the anomalies, and puzzle pieces begin to align, revealing an unexpected image: a microbial, swarm-like creature, previously unknown, highly intelligent, and seemingly bent on haunting humanity in retribution for the damage inflicted upon deep-sea ecosystems. While the existence of this creature and the agency attributed to it lie at the heart of the novel’s speculative dimension, the yrr—as the scientists call it—also functions as an allegory for our current understanding of deep-sea life. Being previously unknown, out of sight, and microbial, the yrr represents many of the recently discovered, symbiotic aspects of deep-sea ecosystems, particularly the intricate interplay between microbial and macrobial life forms in extreme environments like hydrothermal vents. Rather than being haunted by a monstrous ‘other,’ the scientist protagonists are confronted with their own oversight: “Oh, God, how stupid can you get?” one of the scientists shouts. “I must be blind! … Even Roche hadn’t noticed that the jelly-like substance was still clearly visible through the microscope” (p. 633). Failing to see the microbial is nothing new in marine science, we learn: “For centuries, people had been too distracted by all the fish, marine mammals, and crabs in the oceans to see the other ninety-nine percent of life” (p. 634). The importance of seeing and understanding these previously unseen microbial worlds is underscored: “The oceans weren’t ruled by sharks, whales, or giant squid, but by legions of microscopic organisms” (p. 634). From this discovery, the connection to the significance of microbial life in general is easily made: “Small organisms are far more likely to survive during periods of mass extinction … They can even survive for thousands of years in a death-like state and come back to life as though nothing had happened” (p. 677). We, humans, in turn, can only live *because of* microbial life: “Our supply of chemicals—oxygen, nitrogen, phosphorus, sulphur, carbon dioxide and so on—depends on the activity of microbes” (p. 677)—and deep-sea microbes are by no means an exception to this rule.

At the end of the story, Johanson discovers that the U.S. Army and CIA are secretly planning an operation to wipe out the Yrr, using the scientists only for their insights into the creature. Johanson is outraged: “Exactly how deluded are you? You might think you rule the world, but if you were to go around exterminating microbes, you’d kill this planet … Humans only survive on this planet because Earth is ruled by microbes” (p. 775). The Yrr, in other words, demands a fundamental rethinking of humanity's position in the web of life, along with a reconsideration of environmental ethics. Difference should not lead to indifference, the scientists argue, but fear of the alien obstructs this view: “We’ll only be intent on destroying them because they’re not the same as us—and we don’t want them creeping into our caves to steal our children” (p. 674). The scientists also critique popular portrayals of the alien. “You all know the movies,” one of them tells the U.S. Navy staff: E.T., Alien, Independence Day, The Abyss, Contact, etc. These films are either about monsters or the sacred: “A superior intelligence descends from space to lead the worthy to a better, brighter future. For lots of people, that’s a comforting thought, but doesn't it remind you of something?” (p. 580). These movies have a religious dimension, the scientist argues, which blinds us: “They’re not really alien, in so far as they just live up to our notions of evil. The movies don’t allow their aliens to be genuinely different. Good and evil are human concepts, and stories that try to do without them seldom catch on. … To communicate with an alien intelligence, we have to understand that” (p. 580). This critique of how the ‘alien’ is depicted in science fiction aligns with the novel’s challenge to how deep-sea life is portrayed in contemporary culture. By depicting the deep as an alien place full of monstrous creatures, we neglect the understanding of what is truly ‘down there.’ Schätzing has no qualms for self-irony as the last scene in which one of the protagonists goes down into the abyss involves—or at least resembles—a divine revelation: “All that Weaver can discern at this moment is …. Beauty … Weaver doesn't believe in God, but she has to stop herself lapsing into prayer” (p. 861). Weaver, however, drags herself back into reality: It is no Godly creature that reveals itself, only the luminous interplay of unicellular organisms. This ‘all too earthly alien,’ whose ‘playful exploration’ of the submarine evokes feelings of paralyzing beauty, is only ‘alien’ in the eyes of the beholder.

We may overlook important ‘agents’ in the web of life, such as microbial worlds or large fungal networks. Seeing the yet unseen is crucial for a more profound understanding of the networks we inhabit. The last chapter of the book called ‘contact’ reads as a journey into a new worldview that acknowledges human entanglement in microbial worlds. Weaver, diving to the depths in a submarine to encounter the Yrr, fully surrounded by the dark of the deep, sinks into contemplation. The submarine dives into a dark space full of mysteries, an ‘inner space’ (Schätzing, 2006, p. 953). It is a journey into the depths of the unknown: “Our eyes are wide open, and yet we are blind. Look into the darkness, Karen. Look at what lies at the heart of the Earth. It's dark” (p. 847). Weaver reevokes a scene from her childhood, remembering herself walking down a street. She sees a man, a woman walking a dog. She counts four beings: the dog, the man, the woman and herself. Then she ‘sees’ all the other creatures swarming in the snapshot. She sees the trees, the birds, the plants. But even this is only a small fragment of all the living creatures inhabiting her memory snapshot: “Wrong!” she calls out, “What you don't see is that billions of organisms are swarming all over the street” (p. 849). Now, she also sees the mites inhabiting the dog’s fur and her pores, the fleas, the ticks, the mosquitoes, the worms in the intestines, the bacteria, the spores and the viruses: “Floating above the street are spores, bacteria and viruses that form chains of organic matter—chains of which humans are a part—knitting us together in one super-organism” (p. 850). She struggles to grasp the scales of time and space she inhabits: “To understand the world, you need to find a different way of seeing time … Our minds are still bounded by a temporal horizon that prevents us seeing any further than a few years in either direction” (p. 850). Failing to see the consequences of its actions, humanity is striving towards the abyss of its own existence. The Yrr, however, remembers everything. The creature constantly duplicates its genomes along with all the information. New cells do not inherit abstract knowledge but immediate experiences. They have a collective memory; every particle of the larger creatures remembers; and live in all timescales at the same time (p. 758). We humans, Crowe argues, only see a small fragment of the whole. That is why we destroy the planet, failing to see the consequences. Humanity betrays the cause of the world, Johanson argues, by creating a misunderstanding between life forms and their importance: “We judge nature by what we see, but we see only a fraction of it” (p. 677). To see the unseen, then, should be central to any narrative.

## Conclusion

The central concern of this paper was to address the significant discrepancy between present-day scientific knowledge and cultural narratives of the deep, with a particular focus on the narrative representation of deep-sea microbes. Three predominant narratives concerned with the deep have been discussed: that of the deep as a barren place, as a place full of alien life, and as the last wilderness/frontier on earth. The idea of the deep as a barren place has been clearly invalidated by modern scientific exploration and only remains persistent in a mixed form, namely, that of the deep-sea as a vast desert with islands full of biodiversity. The narrative of the deep as a place full of alien life continues to penetrate the cultural imaginary, perpetuating problematic ethical taxonomies. The narrative of the deep as the last wilderness/frontier on earth is a relatively new phenomenon dwelling in the epistemologies of the Anthropocene. Due to its richness in minerals, it might become an increasingly industrialized area, and the last commodity frontier will be stretched with unknown but potentially devastating consequences. Instead of recycling problematic narratives, *The Swarm* articulates the relevance of deep-sea ecosystems to the Earth system and captures human entanglement in ‘alien’ worlds while problematizing and deconstructing its alienness at the same time. This profound engagement with microbial alterity positions the microbe as an important actor in human lifeworlds instead of allowing it to reside at the fringes of the cultural imagination. The global pandemic of COVID-19 has reminded us of the deep entanglements between humans (hosts), animals (transmitters) and microbes (viruses); at the same time, however, its accompanying narratives may only retell a story of germs and diseases—leaving little room for articulating our dependence on the functioning of the global microbiome. Counternarratives are essential for a profound and sophisticated understanding of the microbial world, shedding light on the yet unseen and unknown. And when deep-sea science has made one thing clear, it is that there may be more residing in the dark than previously assumed.

## Footnotes

1 According to NOAA, the national ocean research organization of the United States, more than eighty percent of the ocean is “unmapped, unobserved and unexplored” (NOAA, 2025). Only approximately 0.05 percent of the ocean floor has been mapped at 7 m resolution by underwater vessels (Copley, 2014).

2 I use the terms ‘deep-sea,’ ‘deep ocean,’ and ‘the deep’ interchangeably throughout the text to avoid repetitive rephrasing. They all generally refer to the same: the part of the ocean beyond 200 m in depth (i.e., the bathyal, abyssal, and hadal zones).

3 Ernst Haeckel famously believed that the deep-sea contained a kind of substance that could serve as a missing link between inorganic matter and organic life. Thomas Henry Huxley discovered coccolithophores thriving at a depth of 800 m and speculated that it might be Haeckel's primordial slime. However, it turned out to be an alcohol precipitate of organic debris (Jannasch & Taylor, 1984, p. 487).

4 This is my own definition, informed by (Langley, 2013; Valk & Sävborg, 2018).

5 The Alvin expedition revealed the existence of archaea, a third domain in the tree of life, recognized as the closest prokaryotic relative to eukaryotes (Embley & Williams, 2015), which hinted at hydrothermal vents as potential sites for abiogenesis (Jordan et al., 2019). The discovery of microfossils in hydrothermal vents further solidifies these extreme environments as potential cradles for the earliest life forms known to us (Dodd et al., 2017).

6 Ancient nonactive hydrothermal sites have been found on Mars and active systems are most likely to be found on Jupiter's moon Europa (Michalski et al., 2017).

7 For a comprehensive cultural anthropological study of this ‘microbial turn’ in marine studies, see Stefan Helmreich’s *Alien Ocean* (2009).

8 The term ‘meme,’ first introduced by evolutionary biologist Richard Dawkins in his 1976 book *The Selfish Gene*, describes a unit of cultural transmission or imitation, analogous to a gene in biological evolution. While any analogy between culture and biology warrants critical scrutiny (cf. Boyd & Richardson, 2024; Dupré, 2004), the concept is useful for illustrating the relatively uncontroversial idea that cultures comprise numerous small elements that evolve, interact, and adapt over time.

9 Aligning closely with, or being equivalent to, ‘traveling concepts’ (Bal 2002; Baumbach et al., 2012) or ‘traveling forms’ (Levine 2015).

10 As Scott Atran argues, in most folk ontologies, creatures lacking ‘phenomenal resolution’ are easily lumped together in a residual category, dismissed without further investigation and engagement (1993, p. 32).

11 This is the initial German publication date. Throughout this essay, I rely on the English translation by Sally-Ann Spencer, which was published in 2006 by HarperCollins Publishers Inc.
